# *mleS* in Staphylococcus aureus Contributes to Microaerobic Metabolic Activity, Abscess Formation, and Survival in Macrophages

**DOI:** 10.1128/spectrum.00909-23

**Published:** 2023-04-13

**Authors:** Fengning Chen, Yuyao Yin, Hongbin Chen, Shuguang Li, Guankun Yin, Hui Wang

**Affiliations:** a Department of Clinical Laboratory, Peking University People’s Hospital, Beijing, China; b Institute of Medical Technology, Peking University Health Science Center, Beijing, China; China Agricultural University

**Keywords:** NAD^+^-dependent malolactic enzyme coding gene (*mleS*), *Staphylococcus aureus*, macrophage survival, metabolism, mouse skin abscess

## Abstract

Staphylococcus aureus is subdivided into lineages termed sequence types (STs), infections of which necessitate the expression of virulence factors and metabolic adaptation to the host niche. Given that mechanisms underlying the dynamic replacement of sequence types in S. aureus populations have yet to be sufficiently determined, we investigated the role of metabolic determinants in epidemic clones. *mleS*, encoding the NAD^+^-dependent malolactic enzyme, was found to be carried by the epidemic clones ST59 and ST398, although not by ST239 and ST5. The genomic location of *mleS* in the metabolism-associated region flanked by the thiol-specific redox system and glycolysis operon implies that it plays significant roles in metabolism and pathogenesis. Mouse skin abscess caused by the BS19-*mleS* mutant strain (isogenic *mleS* mutant in an ST59 isolate) was significantly attenuated and associated with reductions in interleukin-6 (IL-6) and lactic acid production. *mleS* deletion also impaired S. aureus biofilm formation and survival in RAW264.7 cells. The BS19-*mleS*-mutant was also characterized by reduced ATP and lactic acid production under microaerobic conditions; however, NAD^+^/NADH levels remained unaffected. *mleS* is thus identified as an epidemiological marker that plays an important role in the microaerobic metabolism and pathogenesis of epidemic S. aureus clones.

**IMPORTANCE** Given the importance of metabolic adaptation during infection, new insights are required regarding the pathogenesis of S. aureus, particularly for epidemic clones. We accordingly investigated the role of metabolic determinants that are unique to the epidemic clones ST59 and ST398. Our results provide evidence that the NAD^+^-dependent malolactic enzyme-coding gene *mleS* is an epidemiological marker that plays an important role in the microaerobic metabolism and pathogenesis of epidemic S. aureus clones.

## INTRODUCTION

Although it is a common human skin and mucosae commensal bacterium, Staphylococcus aureus is also a notable pathogen that causes infections ranging from mild skin infections to fatal diseases (1–4). The clinical presentation of S. aureus infections is determined based on a diverse repertoire of virulence and metabolic factors produced by the major lineages (sequence types [STs]) (5, 6), including the epidemic clones ST59 and ST398. These clones are proficient at expressing core genome toxin genes, such as *psmα* and *hla* (7), and we previously identified *chp* as a strong candidate for the increased cytolytic potential of ST59 (6).

In addition to expressing virulence factors, S. aureus can modify its metabolism and thereby enhance the efficiency of colonization and pathogenesis (8). For example, the arginine catabolic mobile element has been shown to combat host polyamines, thus promoting skin colonization by S. aureus strain USA300 (9). Recently, we identified the NAD^+^-dependent malolactic enzyme-coding gene *mleS* as being unique to the genomes of ST59 and ST398.

Malolactic fermentation (MLF) is a widely investigated process in lactic acid bacteria which involves the decarboxylation of l-malate to l-lactate and carbon dioxide via catalysis of the malolactic enzyme (MLE) (10), thereby resulting in the generation of a high proton motive force, which in turn can drive ATP synthesis via the F_0_F_1_-ATPase (11). Reportedly, *mleS* is associated with the bile stress response in lactic acid bacteria (12), as well as enhancing bacterial viability under environmental stress conditions, such as low pH, oxidative stress, and starvation, in Streptococcus mutans (13, 14). Additionally, YtsJ has been found to catalyze NADP^+^-dependent malate decarboxylation, as well as to generate a transhydrogenation cycle that ultimately involves the conversion of NADPH to NADH, thereby facilitating the rapid adjustment NADPH/NADP^+^ levels in Bacillus subtilis and Escherichia coli in response to cellular requirements (15). Moreover, malic enzymes have been shown to be essential for the intracellular survival of Mycobacterium tuberculosis (16).

Functional annotation of *mleS* has indicated its potential roles in microaerobic metabolism. ST59 and ST398 are frequent causes of skin and soft tissue infections, and skin abscess formation has been established to be associated with a reduction in oxygen levels (17, 18). The invasion of skin by S. aureus is mediated by virulence factors and host tissue damage and is also accompanied by a reduction in oxygen and the availability of glucose (17, 19), thus highlighting the fact that the tissue invasion and persistence of S. aureus necessitate elevated levels of microaerobic metabolism (20). The importance of metabolic adaptation during infection (21, 22), and the as yet undetermined contribution of *mleS* to bacterial pathogenesis, prompted us to investigate the role of this gene in S. aureus pathogenesis.

To evaluate the potential involvement of *mleS* in S. aureus pathogenesis and metabolic activity, we conducted a series of metabolic and virulence experiments using clinical wild-type and *mleS*-mutant methicillin-resistant strains of S. aureus. These included assessments of mouse skin abscess formation capacity, survival in murine RAW264.7 cells, and metabolic activity under microaerobic conditions. The results obtained provided evidence to indicate that *mleS* is involved in enhancing mouse skin abscess formation accompanied by increases in the production of interkeukin-6 (IL-6) and lactic acid, as well as promoting survival within host macrophages. In addition, we established that *mleS* facilitates lactic acid and ATP production under microaerobic conditions.

## RESULTS

### *mleS* is an epidemic clone marker.

**FIG 1:**
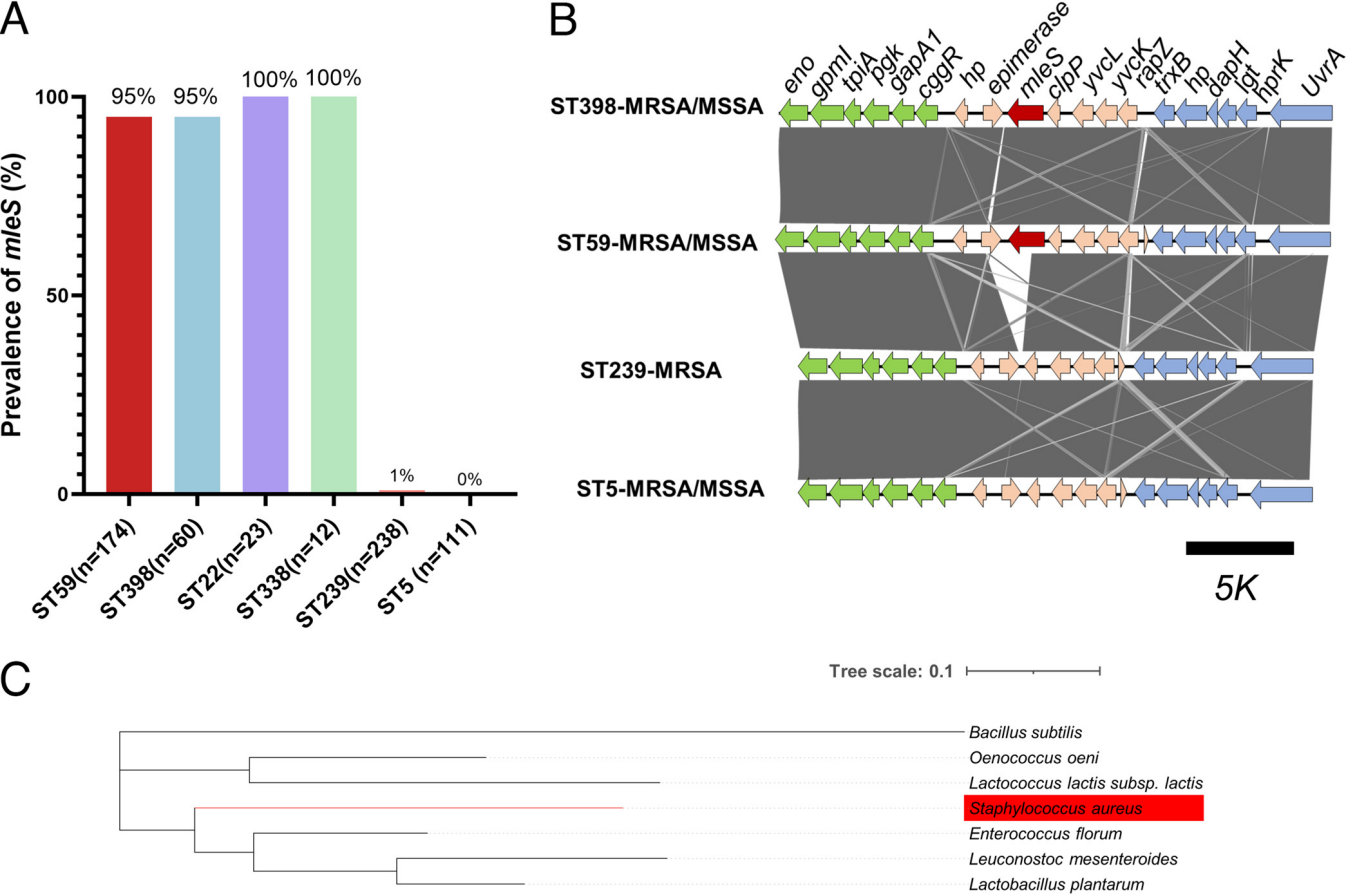
*mleS* is prevalent in the epidemic clones ST59 and ST398. (A) The prevalence of the *mleS* gene in Staphylococcus aureus epidemic isolates. (B) A schematic representation of the *mleS* region among the different clones, generated using Easyfig. Green, glycolytic-associated gene cluster; light blue, thiol-specific redox system; pink, other genes; red, *mleS*. (C) Clustal Omega alignment of *mleS* protein sequences showing the marked differences in different species.

Within the S. aureus genome, *mleS* was annotated as an NAD^+^-dependent malic enzyme-encoding gene. A blastn search with identity and coverage of >95% was applied against our S. aureus strain collection, and the results showed that the overall prevalence of *mleS* in the assessed strains (*n* = 847) was 31.5% (267/847). The prevalence of *mleS* in the ST59 and ST398 clones was 95% and in the ST22 and ST338 clones was 100%, whereas we obtained only a 0% to 1% detection rate for the ST5 and ST239 clones (Fig. 1A). Furthermore, we investigated the genetic environment of *mleS* among the different S. aureus clones, based on an analysis of extracted DNA fragments from the upstream and downstream regions of *mleS*, which were compared using the Easyfig genome comparison visualizer. Unlike *Lactobacillus* spp., in which the malic enzyme and malolactic enzyme pathways are functionally linked in an operon (23), in S. aureus, rather than being grouped in a cluster, the respective gene is instead located in close proximity to the metabolism-associated region, flanked by the thiol-specific redox system and glycolysis operon (Fig. 1B). Clustal Omega alignment of *mleS* protein sequences revealed marked differences between different species, with the *mleS* sequence in S. aureus being most similar (67.9% identity) to one of the putative NAD^+^-dependent malic enzymes in Enterococcus florum (Fig. 1C).

### *mleS* regulates metabolic activity under microaerobic conditions.

A CRISPR RNA-guided cytidine deaminase (pnCasSA-BEC) base-editing system was applied to construct the *mleS* mutant strain in a representative clinical ST59 methicillin-resistant S. aureus (MRSA) isolate, and there is no additional selection marker involved in the base-editing system. Moreover, whole-genome sequencing of the *mleS* mutant and wild-type strains was performed; the results showed that successful mutation in the target site of *mleS* caused a premature stop at codon 25, and no additional mutant was found at other sites in the genome.

**FIG 2:**
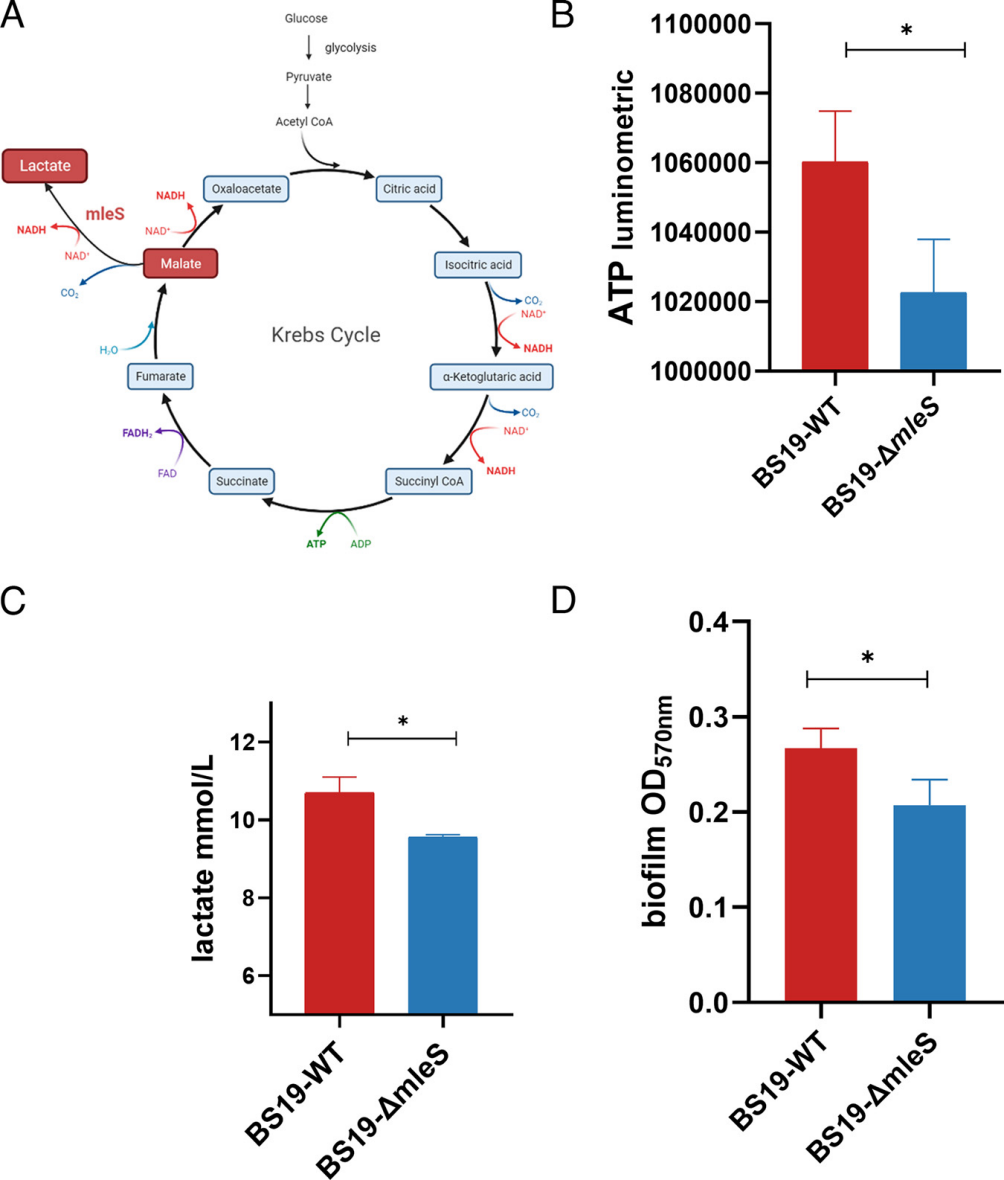
Metabolic functions of the malolactic enzyme. (A) Overview of the malolactic enzyme in the metabolism pathway. (B) Bacterial metabolites were measured under microaerobic conditions. ATP production in the wild-type and *mleS*-mutant, Luminescence was normalized to the OD_600_. (C) Lactic acid in supernatants derived from Staphylococcus aureus cultures was measured using a GEM Premier 3500 analyzer (Werfen, Spain). (D) Biofilm development was assessed by determining the OD_570_ value of crystal violet-stained biofilms. Statistical significance of ATP production, lactic acid production, and biofilm formation capacity were determined using an unpaired Student’s *t* test. Data are shown as the means ± SD of at least three biological replicates. *, *P < *0.05; ns, not significant (*P* ≥ 0.05).

A schematic overview of central carbon metabolism (10, 11, 16) and MLF indicated that *mleS* is associated with lactic acid production and energy metabolism (Fig. 2A). Given that *mleS* is located in close proximity to an operon associated with the glycolytic pathway (Fig. 1B), we examined whether *mleS* plays a role in glycolysis or MLF under microaerobic conditions. The *mleS* mutant strain was observed to have slightly lower ATP production than the wild-type strain (Fig. 2B; *P* = 0.0121). A reduction in lactic acid levels was also observed in the *mleS*-mutant strain (Fig. 2C; *P = *0.0367), thereby providing evidence that *mleS* might be involved in lactic acid biosynthesis, namely, malolactic fermentation. However, we detected no significant differences between the wild-type and *mleS* mutant strains with respect to pyruvic acid levels (see Fig. S1 in the supplemental material), which thus tends to indicate that *mleS* is not associated with glycolysis in S. aureus. After incubating cultures at 37°C without shaking for 24 h, the wild-type strain also exhibited a higher biofilm formation capacity (Fig. 2D; *P* = 0.0121). Furthermore, to verify the association between *mleS* activity and the bacterial cellular redox state, we compared NAD^+^/NADH ratios in the wild-type and *mleS* mutant strains, although no significant differences were detected (Fig. S2). Collectively, these *in vitro* findings indicate that under microaerobic conditions, *mleS* plays roles in the regulation of metabolic activities such as ATP and lactic acid production in S. aureus, which might be associated with its MLF activity.

### *mleS* mutant exhibit impaired survival in macrophages.

**FIG 3:**
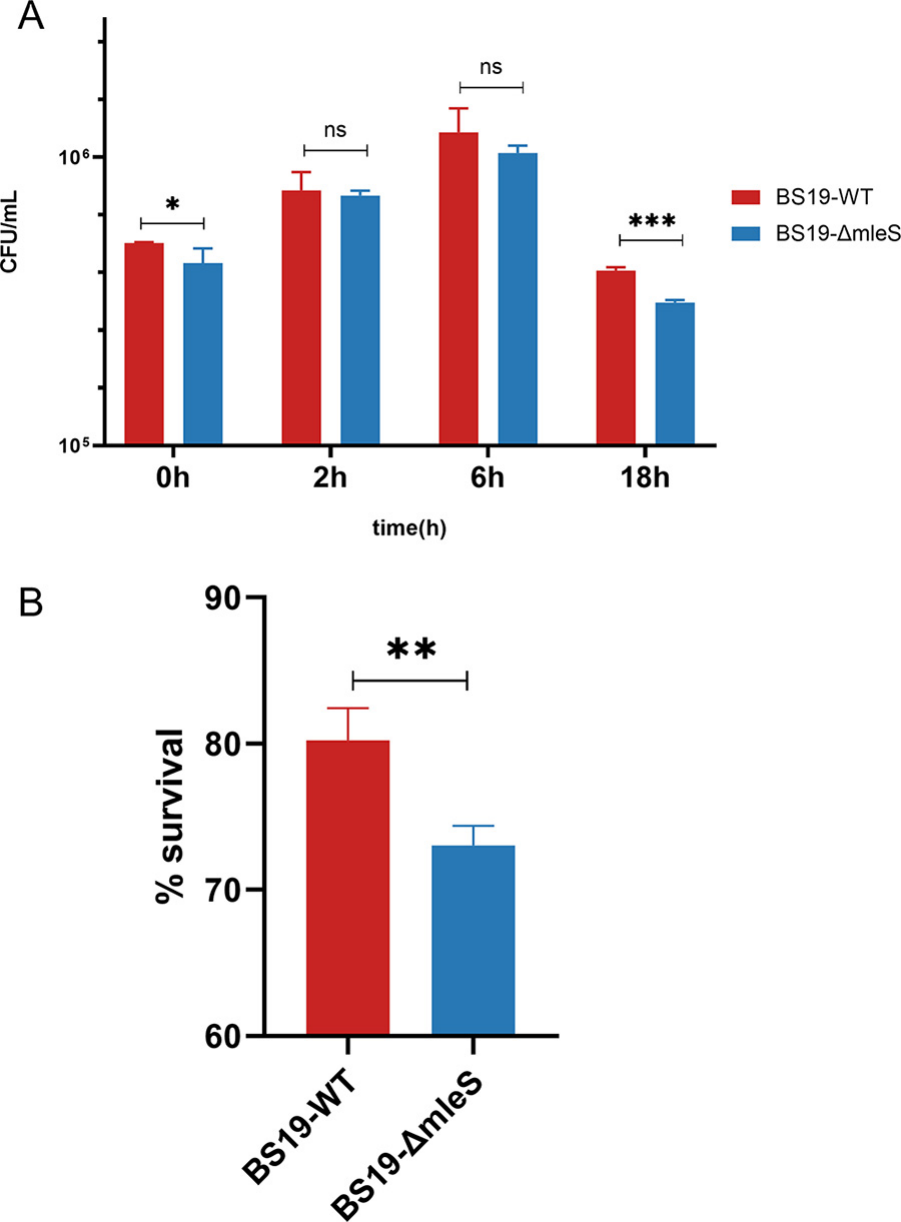
Survival of Staphylococcus aureus in murine RAW264.7 cells. (A) Activated RAW 264.7 macrophages were infected with the wild-type and *mleS* mutant strains at an MOI of 10:1. At 2, 6, or 18 h postinfection, extracellular S. aureus was killed using gentamicin-supplemented culture medium, and the surviving bacteria were enumerated on TSA plates. (B) Percentage of S. aureus survival in activated RAW 264.7 macrophages at 18 h. Student’s *t* test was applied to compare the statistical significance of the survival rate in macrophages. Data are shown as the means ± SD of three biological replicates. *, *P < *0.05; **, *P* < 0.01; ***, *P* < 0.001; ns, not significant (*P ≥ *0.05).

It has been established that malic enzymes are essential for the intracellular survival of M. tuberculosis (16), and we accordingly hypothesized that *mleS* might play a similar role in the intracellular survival of S. aureus. To test this hypothesis, we compared the survival of the BS19-*mleS* mutant and wild-type strains in RAW264.7 murine macrophages, using a gentamicin protection assay. The results revealed that whereas both the *mleS*-mutant and wild-type strains could replicate within 6 h of gentamicin treatment (Fig. 3A), after incubation for 18 h, the *mleS* mutant strain was characterized by significantly reduced survival rate (Fig. 3B; *P* = 0.0002). Although bacterium-derived lactate has been shown to influence the activity of immune cells (24), we detected no difference in lactate production in the supernatants of macrophages co-cultured with the *mleS* mutant or wild-type strains of S. aureus (data not shown). To assess the impact of *mleS* on the virulence factors of S. aureus, we performed transcriptome analysis of the wild-type and *mleS* mutant strains under microaerobic conditions, the results of which indicated that the gamma-hemolysin-coding genes *hlgA* and *hlgC* were downregulated with a fold change ratio lower than 0.5 and a *P* value lower than 0.05 (Table S1). However, given the comparable cytolytic capacities of the wild-type and *mleS* mutant strains, it would appear that *mleS* plays no significant role in the cytolytic activity of S. aureus (Fig. S3).

### *mleS* promotes abscess formation in mouse skin.

**FIG 4:**
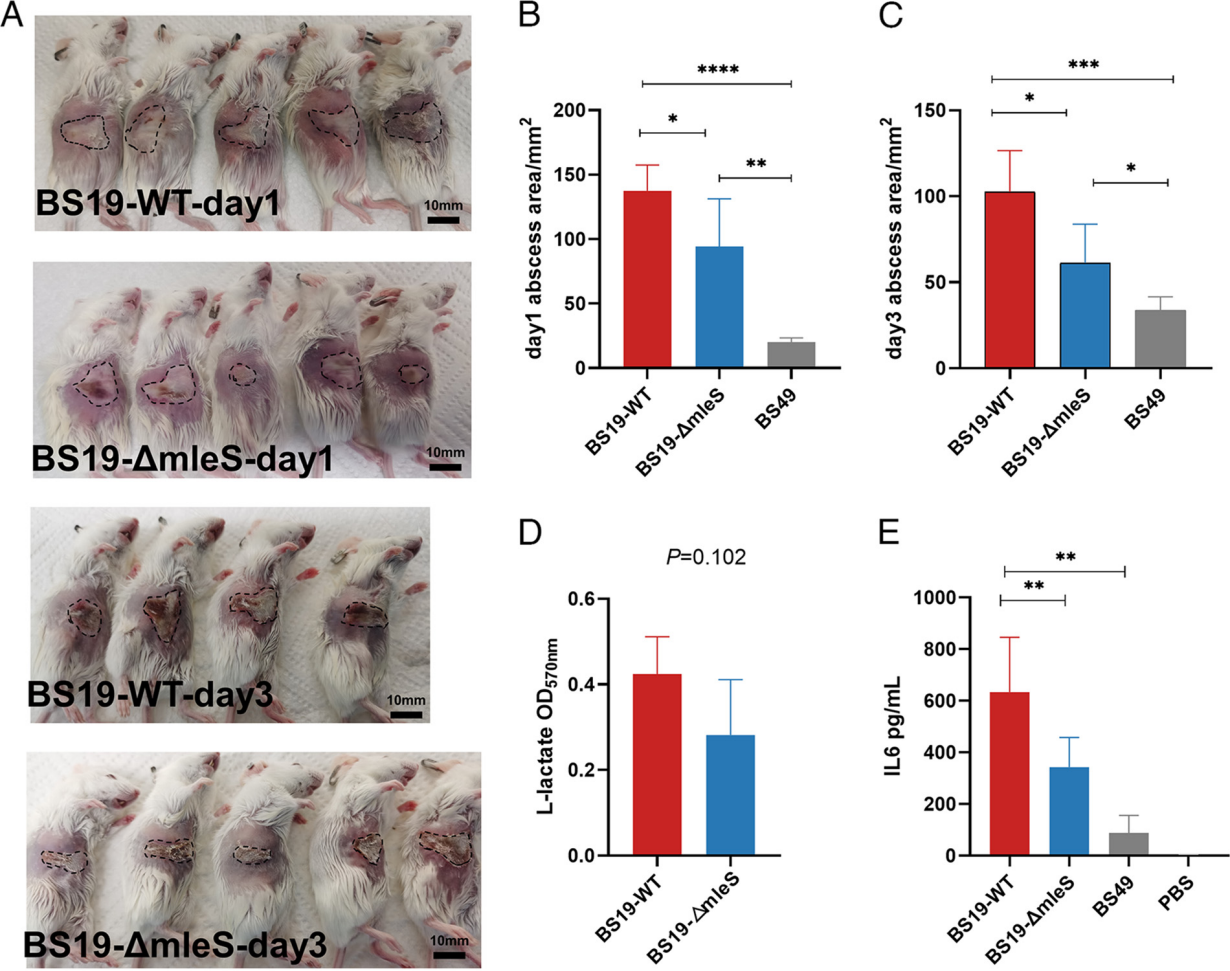
*mleS* in ST59 promotes mouse skin abscess formation. Mice were inoculated via subcutaneous injection with 100 μL of PBS containing 10^8^ CFU of BS19-WT, BS19-*mleS*-mutant, BS49, or PBS alone as a control. (A to C) Representative abscesses at 1 or 3 days after infection. One mouse in the wild-type group failed to develop skin abscesses at day 3 and was therefore excluded from further analysis. The abscess area was measured using a caliper. (D) l-Lactic acid concentrations in infected mouse skin tissue homogenates were determined using a colorimetric assay kit. (E) Cytokine analysis of IL-6 in homolyzed skin tissues using LEGENDplex (BioLegend, San Diego). l-Lactate and IL-6 production was determined using an unpaired Student’s *t* test. Data are shown as the means ± SD of at least three biological replicates. *, *P < *0.05; **, *P* < 0.01; ***, *P* < 0.001; ****, *P* < 0.0001; ns, not significant (*P ≥ *0.05).

ST59 and ST398 have been demonstrated to be frequent causes of skin and soft tissue infections, and in this context, *mleS* serves as an epidemic clonal marker for ST59. Therefore, we used a mouse skin abscess model to assess the function of *mleS* in this regard. Five BALB/c female mice were subcutaneously administered BS19, BS19-*mleS*-mutant, or BS49 (a typical clinical ST239 MRSA isolate used as a low-virulence control) strains. At 1 and 3 days postinfection, reductions in the extant skin lesions were observed in mice with *mleS*-mutant infections compared with those with the wild-type infections (Fig. 4A to C; *P = *0.03 for both groups). These reductions in abscess size were found to be correlated with lower levels of proinflammatory cytokines, such as IL-6 (Fig. 4E). For granulocyte-macrophage colony-stimulating factor (GM-CSF), although the differences were statistically nonsignificant, we detected a reduced trend in the *mleS* mutant strain compared with the wild-type strain (Fig. S4). However, in the case of the other assessed cytokines, IL-1β, tumor necrosis factor-α (TNF-α), gamma interferon (IFN-γ), and IL-27, we observed no significant differences between the wild-type and *mleS* mutant strains.

Among the three treatment groups, we detected comparable numbers of bacteria in the infected skin of mice (Fig. S2A and B) and thus speculated that the observed reductions in abscess size were not attributable to differences in bacterial counts but were instead associated with other factors, such as altered metabolic processes in the *mleS* mutant strain. Moreover, we detected a trend of reduced production of l-lactic acid in the *mleS* mutant strain, although the differences were not statistically significant (Fig. 4D), which thus indicates that certain, as yet undetermined, mechanisms are involved in the attenuation of abscess formation. Collectively, however, our findings indicate that *mleS* contributes to abscess formation in mouse skin, which is associated with reductions in the production of IL-6 and lactic acid, the underlying mechanisms of which await further investigation.

## DISCUSSION

In this study, we demonstrated how *mleS* influences bacterial metabolism and host immune responses to promote skin infections. Our findings indicate that *mleS* is involved in enhancing skin abscess formation and that this process may be associated with increases in IL-6 and lactic acid production and the promotion of S. aureus survival within macrophages. We also provide additional evidence indicating that *mleS* contributes to enhancing ATP and lactic acid yields in S. aureus under microaerobic conditions.

Given that *mleS* is annotated as an NAD^+^-dependent malic enzyme-encoding gene, it is reasonable to assume it might play some roles in influencing the MLF process in S. aureus, thereby contributing to S. aureus adaptation to the skin abscess niche, which is typically a hypoxic environment. In line with these expectations, we found that the production of lactic acid and ATP was impaired in the *mleS* mutant strain under microaerobic conditions. Moreover, the *mleS* mutant strain was shown to be characterized by a reduced biofilm formation capacity, which is consistent with previous findings indicating that lactic acid may contribute to S. aureus aggregation and biofilm formation (25, 26). As bacteria in the innermost regions of biofilms typically experience low oxygen bioavailability, hypoxia is of particular relevance in the context of biofilm infections. However, although we established that the microaerobic metabolic activity of S. aureus was only slightly affected by *mleS*, our data did show a statistical significance. This can probably be ascribed to the fact that the functional loss of *mleS* would be compensated for by other enzymes capable of regenerating ATP, lactate, or NADH under hypoxic conditions.

Whereas malic-associated enzymes have been studied extensively in eukaryotes, the role of MLE in pathogenic bacteria remains poorly understood. Although it has been established that the malic enzyme contributes to the intracellular replication of M. tuberculosis (16), it does not appear to be required for this process in Coxiella burnetii within THP-1 cells (27). S. aureus is regarded as a facultative intracellular bacterium that can survive and replicate within phagocytes (28), and its survival during phagocytosis may be glycolysis dependent (17). Our observations confirming the reduced survival of the *mleS* mutant strain in macrophages compared to the wild-type strain thus provides evidence to indicate that by participating in the MLF pathways within macrophages, *mleS* might promote the intracellular survival of ST59, which may in turn contribute to establishing persistent infections and facilitate dissemination within host (17, 24, 28). Given that we detected no apparent differences between the *mleS* mutant and wild-type strains with respect to virulence genes, we hypothesize that metabolites such as lactic acid might alter the immunometabolism of macrophages, thereby contributing to S. aureus survival and replication (29); however, this assumption necessitates further investigation.

The high prevalence of *mleS* in ST59 and ST398 would tend to indicate that this gene plays a role in host adaptation, particularly during the infection of skin and soft tissues. In this regard, our findings revealed that the loss of *mleS* hampered abscess formation in mouse skin when assessed on the first and third days of infection, which is consistent with our observations of increases in the production of the inflammatory cytokine IL-6 in wild-type BS19. IL-6 is a key STAT3-activating inflammatory cytokine that is induced within the initial hours of inflammation and is linked to hypoxia (30, 31). However, given that we detected comparable numbers of bacteria in the skin tissues of mice infected with the wild-type and mutant strains, we speculated as to whether the altered metabolic processes in the *mleS*-mutant strains impaired its pathogenesis within skin abscesses. Although we detected reduced levels of lactic acid in animals infected with the BS19-*mleS* mutant, the reduction was not statistically significant. One plausible explanation in this regard is that the biosynthesis in S. aureus is mainly associated with *ddh*/*ldh1*/*ldh2* (32, 33), and consequently, the malic enzyme activity of *mleS* may play a comparatively minor role in lactic acid generation. Although the mechanisms whereby *mleS* contributes to abscess formation remain to be elucidated, our observations lead us to believe that these are probably associated with the production of IL-6 and lactic acid, which facilitate inflammatory metabolic adaptation (34).

In summary, in this study, we demonstrated that *mleS* served as an epidemiological marker of epidemic clones of S. aureus and played key roles in the microaerobic metabolism and pathogenesis of *S. aureus* epidemic clones. These findings contribute to enhancing our understanding of the coordination between metabolism and virulence, thereby providing new insights for the development of intervention strategies for effectively targeting and treating epidemic S. aureus infections.

## MATERIALS AND METHODS

### Bacterial strains, media, and growth conditions.

The bacterial strains and plasmids used in this study are listed in Table 1. E. coli was grown in lysogeny broth (LB) medium with shaking at 200 rpm or on LB agar plates at 37°C. S. aureus was grown in tryptic soy broth (TSB) with shaking at 200 rpm or passaged on tryptic soy agar (TSA) at 37°C. When required, the culture medium was supplemented with antibiotics (100 μg/mL kanamycin for E. coli and 10 μg/mL chloramphenicol for S. aureus).

**TABLE 1:** Strains and plasmids used in this study

Strain or plasmid	Relevant characteristics	Source
E. coli strains		
Trans1-T1	F-φ80(lacZ)ΔM15ΔlacX74hsdR(rk-, mk+)ΔrecA1398endA1tonA	TransGen
S. aureus strains		
BS19	ST59-MRSA, derived from a clinical isolate from a bacteremic patient	Current study
BS49	ST239-MRSA, derived from a clinical isolate from a bacteremic patient	Current study
BS19-ΔmleS	BS19 *mleS* mutant	Current study
RN4220	Restriction-deficient strain derived from NCTC 8325–4	The toxic shock syndrome exotoxin structural gene is not detectably transmitted by a prophage
Plasmids		
pCasSABEC	S. aureus genome-editing vector, Kmr, Cmr	Highly efficient base editing in S. aureus using an engineered CRISPR RNA-guided cytidine deaminase
pCasSABEC-mleS	pCasSABEC derivative for *mleS* deletion	Current study

To induce hypoxic conditions, overnight cell cultures were transferred to 15-mL Falcon tubes which were completely filled, leaving no residual air bubbles in the tubes. The filled Falcon tubes were then incubated in anaerobic gas-producing bags at 37°C with gentle shaking until the exponential stage of growth.

### Bioinformatic analysis.

blastn was used to determine the prevalence of *mleS* in 847 clinical S. aureus strains. The genetic environments surrounding the *mleS* locus were identified using Easyfig software (35). The MLE protein was subjected to a blast search against the UniProtKB (https://www.uniprot.org/) database to identify similar sequences, and the six closest six sequences were selected based on sequence similarity. Subsequently, a maximum likelihood tree was generated using Clustal Omega in MEGA X (36).

### *mleS* inactivation.

An inactivated *mleS* mutant was generated in the BS19 strain, a representative clinical ST59-MRSA isolate, using a CRISPR RNA-guided cytidine deaminase (pnCasSA- BEC) base-editing system, as previously described (37). Briefly, annealed *mleS* spacer oligonucleotides (Table 1) were inserted into the BsaI sites of a pnCasSA-BEC plasmid using Golden Gate assembly. pnCasSA-BEC plasmids carrying the *mleS* spacer were initially transformed into E. coli strain Trans T1, modified in S. aureus RN4220, and then transformed into the target clinical S. aureus strains via electroporation. Gene mutations were confirmed based on analytical PCR and sequencing, and whole-genome sequencing of the *mleS*-mutant and wild-type strains was performed to confirm the absence of any additional mutants.

### RNA-seq.

Total RNA was extracted from the wild-type S. aureus BS19 and BS19-Δ*mleS* strains cultured under microaerobic conditions using an RNeasy kit (Qiagen). All extracted RNA was subjected to transcriptome sequencing (RNA-seq) analysis, with rRNA being removed using a Ribo-Zero magnetic kit (Zhongbei Linge Technology Development Co. Ltd., Beijing, China). RNA integrity was determined using agarose gel electrophoresis, and purity and concentration were determined using a NanoDrop 2000 spectrophotometer (Thermo Fisher Scientific, USA). An RNA library was constructed using a TruSeq RNA sample preparation kit (Illumina, San Diego, CA, USA) and sequenced using the NovaSeq 6000 platform (Illumina). To assess gene expression levels, for each gene, we calculated fragments per kilobase of exon model per million mapped reads values. A false-discovery rate-adjusted *P* (*P* adj) value of 0.05 and fold change of 2.0 were set as thresholds to identify differentially expressed genes.

### Detection of metabolites.

Bacterial metabolites were measured under microaerobic conditions. Culture supernatants were assessed for bacterial lactic acid production, the content of which was analyzed using a GEM Premier 3500 analyzer (Werfen, Spain). Bacterial ATP production was determined using a BacTiter-Glo microbial cell viability assay (G8230; Promega), as described previously (17). Briefly, 100 μL of bacterial suspension and 100 μL BacTiter-Glo reagent were added to the wells of 96-well plates (3606; Costar). Luminescence was recorded after 5 min of incubation in the dark, and ATP levels were normalized to optical density at 600 nm (OD_600_) values. The NAD^+^/NADH ratio was measured using an NAD^+^/NADH assay kit with WST-8 (S0175; Beyotime). The total amounts of NAD^+^ and NADH were expressed with respect to bacterial protein concentrations using the bicinchoninic acid (BCA) method. To measure bacterial pyruvic acid production, we used a colorimetric assay (BB-47421; BestBio) according to the manufacturer’s instructions, with the content of pyruvic acid being expressed in terms of the bacterial protein concentration. l-Lactic acid concentrations in infected mouse skin tissue homogenates were determined using l-lactic acid colorimetric assays (24) according to the manufacturer’s instructions (ab65330; Abcam). The skin lesions were harvested and weighed, and an equivalent volume of phosphate-buffered saline (PBS) was added to avoid the error effect of cell numbers in the abscess area.

### Biofilm formation.

For determinations of biofilm formation capacity, bacteria were grown in TSB for 5 h at 37°C until reaching an optical density at 600 nm (OD_600_) of 0.6 (indicating the logarithmic phase of growth). Cultures were then diluted (1:100) in TSB-1% glucose (5 mL), inoculated into radioimmunoassay vials, and incubated at 37°C without shaking for 24 h. Biofilm development was assessed by determining the OD_570_ values of crystal violet-stained biofilms as previously described (6). The assay was performed in biological replicates.

### *In vitro* cytolytic assays.

Culture filtrates were used to lyse erythrocytes. Strains of S. aureus were cultured for 18 h in TSB, and culture supernatants were added to human red blood cells (2% vol/vol in phosphate-buffered saline [PBS]), followed by incubation at 37°C for 1 h with gentle shaking. Cells suspended in PBS or TSB were used as blank controls. Cultures were subsequently centrifuged at 1500 × *g* for 10 min at 4°C without disturbing the cells. Aliquots of the resulting supernatants were transferred to the wells of fresh 96-well plates, and hemolysis was determined by measuring the optical density at 540 nm using a spectrophotometer. The assay was performed in triplicate. The statistical significance of cytolytic capacities was determined using an unpaired Student's *t* test.

### RAW264.7 infection.

Bacterial survival following phagocytosis was assessed as previously described (17). Briefly, RAW 264.7 cells (1 × 10^6^ cells/mL) were suspended in Dulbecco’s modified Eagle’s medium (DMEM, Gibco) without fetal bovine serum (FBS), seeded into the wells of a 48-well plate (0.5 m/well), and incubated for 6 h at 37°C, followed by activation via IFN-γ (20 ng/mL). The S. aureus cells were cultured in TSB medium to the mid-exponential phase of growth (OD_600_, 0.6) for macrophage infection. S. aureus cells were washed with PBS and incubated at a multiplicity of infection (MOI) of 10:1 for 0.5 h at 37°C to facilitate phagocytosis. Fresh medium containing 100 μg/mL gentamicin was added for 1 h at 37°C to kill residual extracellular bacteria. The infected cells were washed twice with PBS, after which, 0.02% Triton X-100 was added to certain wells to induce cell lysis, and bacterial enumeration was performed based on dilution plating (*t* = 0). Thereafter, fresh medium containing low-dose gentamicin (12 μg/mL) was added to cells, which were incubated for 2, 6, and 18 h prior to lysis. The assay was performed using three biological replicates.

### Mouse model of subcutaneous abscesses.

For mouse model development, we used outbred immunocompetent female BALB/c mice (6 to 7 weeks old). The hair on the backs of these mice was removed using an animal shaver and depilation cream. For mouse infection, S. aureus cells were cultured in TSB medium to the mid-exponential phase of growth (OD_600_, 0.6). The mice were anesthetized and inoculated with either 100 μL PBS or PBS containing ~10^8^ live S. aureus organisms via subcutaneous injection in the back. Lesion development was monitored daily, and the infected areas were biopsied on the first and third days after infection. The bacterial burden in homogenized biopsy specimens was measured via serial dilution and plating on blood agar plates. To analyze cytokine expression in the skin, mice were euthanized at 72 h after injection, and sections of the skin lesions were harvested for LEGENDplex (740446; BioLegend) cytokine analysis.

### Statistical analyses.

GraphPad Prism 8 was used for statistical analysis. Student’s *t* tests, chi-square tests, or Fisher’s exact tests were performed to determine the statistical significance of the data. For nonparametric data, we used the Mann-Whitney U test. The error bars in all graphs indicate the standard deviation of the mean (mean ± SD). Results with a *P* value of <0.05 were considered statistically significant.

### Ethics statement.

All animal experiments were performed in accordance with the Laboratory Animal Care and Use Guidelines of the Chinese Association for Laboratory Animal Sciences (CALAS). This study was approved by the ethics committee of the Peking University People’s Hospital (2022PHE055).

### Data availability.

The raw sequence data reported in this paper have been deposited in the Genome Sequence Archive (GSA) in the National Genomics Data Center, China National Center for Bioinformation/Beijing Institute of Genomics, Chinese Academy of Sciences (GSA no. CRA009926) and are publicly accessible at https://ngdc.cncb.ac.cn/gsa.
