# Supplementary material for: mleS in Staphylococcus aureus Contributes to Microaerobic Metabolic Activity, Abscess Formation, and Survival in Macrophages
Source: Microbiol Spectr. 2023 Apr 13;11(3):e00909-23. doi: 10.1128/spectrum.00909-23 (PMC10269618; doi:10.1128/spectrum.00909-23)

Fig. S1. Pyruvic acid concentration in wild-type and *mleS*-mutant strains of *Staphylococcus aureus*. The concentration for pyruvic acid is expressed as the bacterial protein concentration. Data are shown as the means ± SD of three biological replicates. NS, not significant ($P \geq 0.05$).

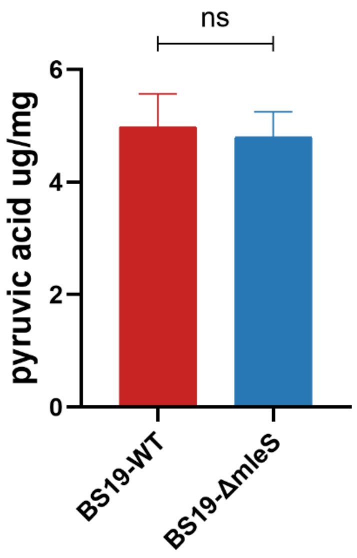

Fig. S2. NAD$^+$/NADH ratio between the wild-type and *mleS*-mutant strains of *Staphylococcus aureus*. Statistic differences were determined by using an unpaired Student's *t*-test. Data are shown as mean ± SD of three biological replicates. NS, not significant ($P \geq 0.05$).

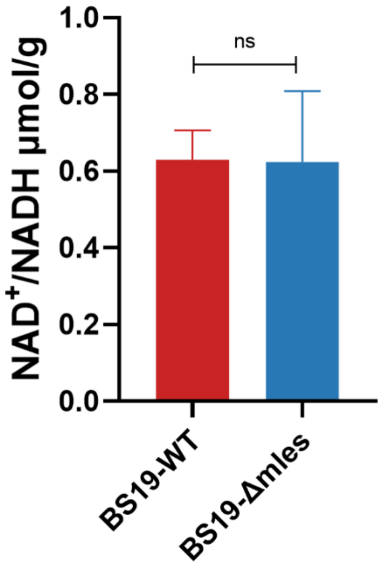

Fig. S3. Cytolytic capacities of the wild-type and *mleS*-mutant strains of *Staphylococcus aureus*. Data are shown as the means ± SD of three biological replicates. NS, not significant ($P \geq 0.05$).

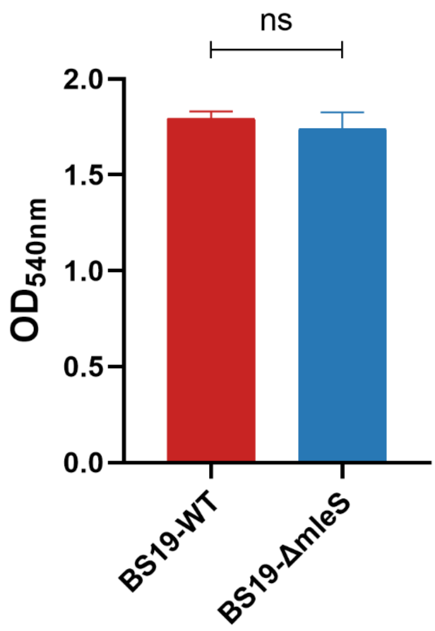

Fig. S4. A, B. Bacterial number in the infected skins day 1 and 3 of infection. **C.** Cytokine analysis of GM-CSF in homolyzed skin tissues using LEGENDplex (BioLegend, San Diego). Statistic difference were determined by using an unpaired Student's *t*-test. Data are shown as the mean ± SD of at least three biological replicates. ** $P < 0.01$; NS, not significant ($P \geq 0.05$).

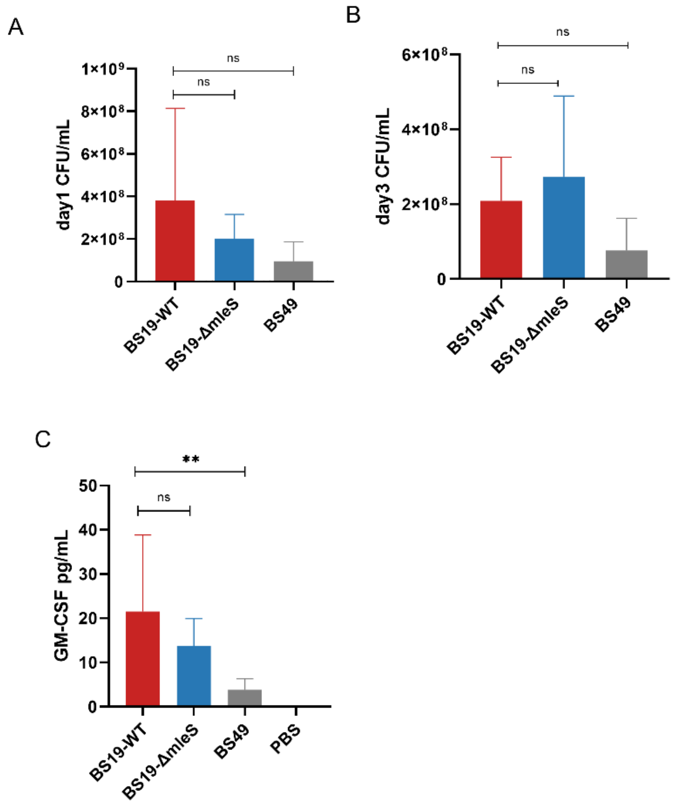

Supplement: Supplemental file 2 — Supplemental material. Download spectrum.00909-23-s0002.pdf, PDF file, 0.3 MB [file spectrum.00909-23-s0002.pdf]
